# The Mediating Role of Organizational Commitment on the Relationship Between Emotional Labor and Work Engagement of Teachers

**DOI:** 10.3389/fpsyg.2021.648404

**Published:** 2021-07-05

**Authors:** Gozde Sezen-Gultekin, Mustafa Bayrakcı, İbrahim Limon

**Affiliations:** ^1^Educational Administration and Supervision Program, Educational Sciences Department, Faculty of Education, Sakarya University, Sakarya, Turkey; ^2^Mithatpaşa Anatolian High School, Sakarya, Turkey

**Keywords:** emotional labor, work engagement, organizational commitment, organizational psychology, teachers

## Abstract

This study aims to investigate the mediating role of teachers’ organizational commitment (OC) on the relationship between their emotional labor (EL) and work engagement (WE). The study employed a cross-sectional design. The sample of the study consisted of the teachers working in Sakarya province of Turkey. They participated in the study voluntarily and responded scale items online. The findings showed that teachers’ perceptions of EL, OC and WE is relatively high. Also, there are statistically significant and positive correlations among variables. On the other hand, the findings confirmed the hypotheses. Teachers’ EL predicts their OC and WE. Additionally, OC predicts WE and plays a mediating role on the relationship between EL and WE. Based on the findings some suggestions were made.

## Introduction

In order to accomplish competitive advantage in the modern world, organizations need human resources with high levels of energy, efficiency, and commitment (Bakker and Schaufeli, 2008; Chen, 2018). Many organizations already realize that positive job-related attitudes such as commitment and engagement are of critical importance in terms of their competitive advantage (Walker, 2001; Chew, 2004). Teachers are not an exception in this sense and are expected to demonstrate strong professional motivation and have a high level of dedication and work engagement (WE). Engaged teachers are completely devoted in their work, entirely committed, and dedicated to it, while actively disengaged teachers are frustrated and dissatisfied with their jobs, perform poorly, and have a negative effect on their co-worker’s efforts in the organizations (Paulík, 2020). Considering the present economic landscape, the competitive advantage of organizations can be improved by an engaged workforce (Hoole and Bonneman, 2015). As WE refers to favorable emotions and motivating energy, engaged workers tend to exhibit behaviors that may result in desired outcomes for organizations. Employees who are actively engaged are inclined to cope better with extreme requirements in the work environment, respond more easily to organizational change, and creatively solve the problems (Bakker and Demerouti, 2008; Othman and Nasurdin, 2012). In recent years, researchers from different academic disciplines and business people from many industries including educational sector have paid considerable attention to WE (Bakker and Demerouti, 2008; Schaufeli, 2013; Breevaart et al., 2015; Demerouti et al., 2015). However, evidence suggests that only 40% of employees were strongly engaged (Towers Watson, 2014). WE is thus still accepted as a very important issue by researchers and business people (Iqbal et al., 2012). So, it is of great importance to investigate the processes through which WE can be boosted.

Studies on WE focused especially on determining its predictors and outcomes (Schaufeli and Bakker, 2004; Kim et al., 2009). In some of these studies WE was associated with personal resources, such as loneliness at work (Sezen, 2014) and self-efficacy (Akhtar et al., 2015; Liu et al., 2017) as well as on job resources, such as team climate and organizational support (Xanthopoulou et al., 2013; Song et al., 2015). On the other hand, there are many studies focusing especially on teachers’ WE mainly based on three reasons. The first one is the relationship of teacher effectiveness and student achievement with WE of teachers. The second is the assumption that engaged teachers generally experience less burnout problems. The third is the belief that engaged teachers tend to be more productive, display organizational citizenship behaviors more frequently and contribute to school (Bakker and Bal, 2010; Klassen et al., 2012).

On the other hand, teachers are emotional workers (Yin, 2015). Teachers’ performance, self-efficacy, job satisfaction, burnout, and instructional effectiveness have been found to be affected by their emotions (Taxer and Frenzel, 2015; Lavy and Eshet, 2018). Since teachers experience complicated relationships with students, colleagues, and parents, emotional labor (EL) plays a fundamental role in teaching. In the school context, EL of teachers can be defined as the requirement to control emotions according to organizational rules and guidelines carrying out the teaching profession (Yin, 2016). Rafaeli and Sutton’s (1987) EL theory suggest that EL effects both the individual and organization. Additionally, Bakker and Demerouti (2008) suggested the job demand resources model arguing that employees’ EL affects psychological well-being individually at first, which in turn affects results at the organizational level. The teaching profession is being gradually regulated by administrative bodies similar to service sector workers who follow the forms of EL (Bolton, 2010). Although the previous studies’ significant contribution to the literature and business management, there are still much to discover in terms of WE and EL in educational organizations.

Moreover, literature suggests that organizational commitment (OC) has a considerable effect on organizational performance. Teachers who have higher OC to their schools have stronger beliefs in the school’s aims and values and prefer to stay in the school (Chan et al., 2008; Meyer et al., 2019). They tend to have a higher motivation to belong to the organization and display organizational citizenship behavior more frequently (Agu, 2015). Employees with a higher commitment experience feeling of warmth, appreciation, and loyalty toward the organization as a result of their positive thoughts and interactions within the organization and have a deep desire to remain within the organization (Casper et al., 2011). Research also found significant relationship between OC and WE.

In the literature, studies mostly focus on the linear relationship between teachers’ EL and WE, or EL and OC (Akın, 2021). However, there is a gap in literature investigating the indirect relationships between these variables which provide insight into processes through which WE can be fostered. Therefore, this study tests a structural model which investigates the relationship between EL and WE and the mediating role of OC on this relationship for teachers.

### Theoretical Background

#### Work Engagement

Positive psychology focuses on the measurable, developable, and manageable strengths and psychological capacity of the human, rather than the negativities such as burnout, conflict, job dissatisfaction in the workplace. For this reason, organizations now look for energetic, dedicated, and focused employees, that is, individuals who are engaged with their work (Sezen, 2014) because such employees are more creative and productive by devoting their abilities and experience to the organization (Bakker and Demerouti, 2008).

Work engagement is described as a positive and fulfilling emotional state about work which is represented by vigor, dedication, and absorption (Schaufeli, 2013). Previous research suggest that the basic elements of WE are dedication and vigor (Schaufeli, 2013; Taris et al., 2017). Employees who have higher levels of vigor and dedication find various ways to manage job demands and obstacles, while achieving psychological and physical health (Mache et al., 2014). Moreover, the absorption dimension, which refers to a complete concentration in work, is often defined by the rapid passing of time or the difficulty of detaching oneself from one’s work (Mauno et al., 2007). According to Schaufeli et al. (2001), vigor refers to the physical power of the body or mind as one works; dedication refers to the emotional condition of the worker, in which he/she has a sense of excitement toward work; and absorption means a cognitive situation in which the individual is engaged to a job with complete concentration (Salanova et al., 2005).

When we consider various definitions in the literature, key features to clarify WE are the positive and exciting motivation that employees show in achieving their job objectives while feeling fully engaged and dedicated in carrying out their job duties (Markos and Sridevi, 2010; Schaufeli, 2013). Teachers’ WE can be regarded as important in terms of overall success of schools because previous research found statistically significant and positive relationships between teacher WE and student achievement (Wilson and Corcoran, 1988; Bakker, 2005). Cardwell (2011) also argued that one of the predictors of student engagement is teacher engagement (cited in Butakor et al., 2020). Engaged teachers achieve more than their formal responsibilities and duties and support their students academically by following various approaches and methods resulting in better a academic performance (Kirkpatrick, 2009; Ariani, 2013).

#### Emotional Labor

Emotional labor is characterized as a term related to proper management of emotions in the workplace and defined by Hochschild (1983) as “the management of emotions to create a publicly observable facial and body display” (p. 7). Ashforth and Humphrey (1993) accordingly represented EL as the attitude of displaying the appropriate feelings. EL can be used as a way to control emotions efficiently in order to achieve organizational goals and to manage attitude or frustration against a coworker or client, which may contribute to improved work efficiency (Grandey, 2000). By this means, Grandey states that EL is about adjusting emotions and remarks to organizational requirements. Jobs which include more physical interaction and therefore require characteristics of EL, include teachers, health care workers, call center workers, etc. (Sloan, 2004; Diefendorff et al., 2006).

Emotional labor strategy may be used, as Hochschild (1983) argues, either with surface acting as altering external appearance to represent required emotions – emotions that are not actually felt privately, or deep acting as modifying their physical expressions in addition to their inner feelings. The surface actor suppresses or conceals his genuine feelings and behaves in a way in that complies with the requirements prescribed by the organization with respect to the emotional displays of its employees. On the other hand, a deep actor adjusts his/her feelings in order to comply with organizational expectations as he/she genuinely feels in any specific circumstance (Lazanyi, 2011). Surface acting requires the simulation of feelings that are not actually felt by the careful display of acceptable verbal or non-verbal signals, either by artificial expression of positive feelings or by suppression of negative feelings (Ashforth and Humphrey, 1993; Grandey, 2000; Diefendorff et al., 2005). Deep acting, on the other hand, requires the real internal modification of negative feelings and attempts to experience the emotion that should be seen (Ashforth and Humphrey, 1993; Diefendorff et al., 2005). In particular, deep acting will occur first and will be preceded by surface acting if it is not appreciated during an interaction (Gross, 1998).

Teaching, as an activity, is an emotional process and teachers repeatedly use emotions in their classroom performance as well as outside the classroom (Deliveli and Kıral, 2020; Töre, 2020). Students prefer teachers who are disciplined but not authoritative, funny but not cynical, and who can pay attention to each student individually and behave equally in punishing and rewarding (Wragg and Wragg, 1998). To establish a thorough classroom atmosphere, positive relationships are required in which teachers should handle, supervise, and adjust their emotions (Zhang and Zhu, 2008; Akın et al., 2014). Teaching is also a demanding job that requires strong human relationships (Genç, 2004) and includes managing frustration and facial expressions in and outside the classroom since repeated declaration of felt tense feelings to students can have a negative impact on the learning process. Teachers are also expected to conform to some tacit organizational intentions for emotional demonstration to students and parents (Berquam, 2020). In addition, monitoring schemes are putting more pressure on them to change their behavior to focus on performing rather than caring aspects in order to pass observations or inspections (Lindqvist et al., 2019). Ineffective emotion control can also negatively affect relationships with colleagues and leaders outside the classroom. Winograd (2003) argued that teaching fulfills Hochchild’s (1983) three criteria that requires EL, which are, (a) face-to-face communication between teachers and others (b) teachers’ producing some emotional state (e.g., joy or fear, and excitement or anxiety); and (c) a degree of external influence over teachers’ EL, which usually comes in the form of cultural expectations or professional norms. Teaching as a profession, therefore, involves the use of a considerable amount of EL (Tösten and Toprak, 2017).

Loh and Liew (2016) observed that teachers perform EL in and outside classrooms; they manage, conceal, and manipulate their emotions in their interactions with students, parents, and colleagues. Emotional display rules in schools and the emotional acting in which teachers engage are embedded within each individual practitioner. Collectively, these rules become a part of the school culture, which in turn plays a significant role in shaping teachers’ conceptions of themselves as professionals in the workplace (Brown et al., 2014). Many teachers consider the performance of emotions which lack in authenticity to be stressful (Ogbonna and Harris, 2004). To facilitate disciplined and orderly classes daily, teachers can incur significant levels of stress considering the amount of EL they experience (Loh and Liew, 2016). This condition has arguably worsened because of numerous demands from schools and students, resulting in a substantial increase in EL over the last 10 years (Taylor, 2020). Considering the previous literature, further examination is needed as teachers today are exposed to ever-increasing levels of EL.

#### Organizational Commitment

Organizational commitment refers to an attitude or psychological condition that characterizes the relationships of employees with their employer and ultimately influences their intentions to stay or leave the organization (Kotzé and Nel, 2020). Numerous definitions of OC can be seen in literature. Bateman and Strasser (1984) suggest that OC involves an employee’s loyalty to the organization, level of aim and value consistency with the organization, and desire to stay within the organization. Commitment is described as a linkage between a person and the organization (Buchanan, 1974) and a psychological condition that characterizes the relationship of employees with the organization with its conclusions to stay within the organization or not (Meyer and Allen, 1997).

There are three dimensions of OC; affective, continuance, and normative commitment (Meyer and Allen, 1997). Affective commitment is the employees’ sense of the emotional attachment with the organization. Continuance commitment specifies employees’ understanding of the cost of leaving the organization. Normative commitment refers to the understanding of the employees of their normal obligation to the organization (Agu, 2015). These three types of commitment are accepted in the literature as a psychological situation that either identifies the relationship of employee with the organization or has the significance to affect whether the employee will stay with the organization or not (Meyer et al., 1993). Research indicates that those with a powerful affective commitment will continue the organizational membership because they want to, with a powerful continuance commitment will continue because they have to, and those with a normative commitment will continue to the organization because they feel that they have to (Meyer et al., 1993).

As for teachers, three types of commitment are introduced in literature: commitment to school, profession, and students. Commitment to school can be defined as the intensity of the identification of an individual to a particular school (Mowday et al., 1979). Commitment to the profession, on the other hand, can be regarded as a positive attachment to teaching. Finally, commitment to students is a teacher’s dedication to student learning (Park, 2015). Previous research shows that teacher’s OC is significantly related to job satisfaction, school performance and self-efficacy (Dee et al., 2006; Park, 2015). Their OC has an important effect on efficacy and success of their work (Fresko et al., 1997; Wang et al., 2020). Teachers with high OC have positive emotions about the mission and ethics of their schools and tend to stay within the school (Hong and Matsko, 2019). OC of teachers is associated with student success and better quality in schools (Nehmeh, 2009; Balay, 2014; Akdemir, 2019). Based on the previous literature, we can say that committed teachers are one of the most important assets of schools.

### Conceptual Framework

#### Emotional Labor and Work Engagement

Although there are many studies in the literature focusing on basic dimensions of EL together, Öztürk (2020), which has been accepted as a basis for this study, proposed a three-dimensional EL model for teachers which is similar to deep acting that perceived EL as a positive drive for teachers. Although most of the studies about EL in the literature are carried out on the service sector employees, Öztürk (2020) investigated teachers’ EL and developed a model of “EL in schools.” Öztürk’s (2020) three-dimensional model of EL in schools comprises “emotional effort,” “emotional transparency,” and “negative emotional transfer” which accepts EL as a positive organizational attitude for teachers as in deep acting.

Previous studies (Chan, 2009; Wang et al., 2010; Heo and Lee, 2015; Kim et al., 2015; Pelosi, 2015; Yoo, 2016; Yoo and Jeong, 2017; Han S. S. et al., 2018; Öngöre, 2019; Košir et al., 2020) found that EL is positively associated with WE. The studies investigating the interaction of EL and employees indicate that deep acting yields positive outcomes (Kim, 2008), and reduces emotional dissonance through a mechanism that makes emotions harmonious to expressions (Richards and Gross, 2000) in terms of WE. Engaged teachers, convinced of their usefulness to the school and the students, are willing to devote their full efforts to their job, despite the difficulty presented by the demands of the profession and the disruptive influences which complicate their work (Paulík, 2020). WE is supposed to foster the positive attitudes or actions of employees toward clients, work, and the organization. The more employees who have WE have sufficient psychological and physical energy levels, the more they display deep emotional activities. So, WE is positively associated with deep acting (Yoo and Arnold, 2014), and accordingly EL is positively associated with WE (Lu and Guy, 2014). Also, EL affects WE positively (Kim et al., 2015; Han S. S. et al., 2018). Therefore, the following hypothesis was suggested.

*H1: Teachers’ emotional labor positively predicts their work engagement.*

#### Emotional Labor and Organizational Commitment

Previous research in the literature indicates that the relationship between commitment and EL are likely to be stronger when they point out similar goals and contexts (Fishbein and Ajzen, 1975; Lavelle et al., 2007). When we consider the correlation between EL and OC, both have been justified to be connected (Xin et al., 2017). Wong and Law (2017) found that performance of EL by employees affects their OC. Moreover, deep acting is specifically based on the employees’ inner feelings (Ashforth and Humphrey, 1993), this type of EL is more consistent with a genuine concern for one’s clients, considering the increased psychic effort involved in deep acting. Deliveli and Kıral (2020) argued that employees’ intentionally presentation of EL to accomplish institutional aims may lead to OC. It may also be inferred that employees who includes emotions into their organizational activities are most likely to stay with that organization meaning that they have higher level of OC (Deliveli and Kıral, 2020).

Lin (2005) found that individual EL has a positive effect on OC and EL plays a mediator role on this relationship (cited in Xin et al., 2017). Furthermore, Zhang and Zhu (2008) had a similar finding regarding the relationship between EL and OC. Öztürk (2020) also proposed EL as a positive organizational variable in his study which is carried out on teachers and accepted as a basis for this study. Society also tests the behavioral performance of teachers using high moral expectations and requires them to act as models to correctly lead students’ learning (Zhang et al., 2020). Additionally, teachers’ commitment is regarded as having an emotional foundation (Berkovich and Eyal, 2017). Meyer et al. (2019) stated that commitment can indicate an emotional connection to particular goals. EL represents one’s emotional management, exhibiting emotions in line with organizational rules and interactions with actors (Hochschild, 1983). Thus, we consider teachers’ commitment is highly affected by their EL in school. Therefore, this study investigates the nature of the association between EL and OC. Based on the previous literature, we developed the following hypothesis.

*H2: Teachers’ emotional labor positively predicts their organizational commitment.*

#### Organizational Commitment and Work Engagement

Previous literature suggests a positive association of WE with well-being (Halbesleben, 2010; Dilekçi and Limon, 2020) and a negative one with turnover intention (Wood et al., 2020). The OC of teachers was identified by Tsui and Cheng (1999) as the relative intensity of their identification with and participation in a specific school. From this point of view, OC of teachers can be characterized by a powerful belief in and recognition of the aims and values of the school, a willingness to utilize noticeable effort on behalf of the school, and willingness to maintain school membership (Sezgin, 2009). While WE was described as a positive state of mind characterized by high energy, excitement, and a complete concentration at work (Schaufeli et al., 2002), OC, on the other hand, was seen as the strength of the identification of the employee with the organization (Mowday et al., 1979; Li, 2014). Saks (2006) emphasizes that while OC reflects attitudes and attachment of employees to the organization, engagement is not attitudinal and thus reflects the focus and absorption of individuals while performing tasks (Santos et al., 2016).

In literature there are different views on conceptualization of the relationship between WE and OC. Some studies hypothesized WE as an antecedent of OC and explored its impact on OC (Hu and Schaufeli, 2011; Albrecht, 2012; Karatepe, 2013); while some others suggested WE as an outcome of OC and investigated the effect of OC on it (Barnes and Collier, 2013; Zhang et al., 2015; Rivkin et al., 2016).

The studies describing OC as an antecedent of WE argue that when employees are attached to their organization, they may demonstrate higher WE (Barnes and Collier, 2013; Zhang et al., 2015). It means OC precedes WE, and when employees are committed to their organizations and willing to pay back to the organization, WE emerges as a kind of repayment (Choi et al., 2015). This view implies that as a result of the attachment of the employees to the organization, attachment to the work occurs, as well. On the contrary, some other studies argue that WE could lead to increased OC (Albdour and Altarawneh, 2014). These studies suggest that when people have WE, they form a connection with work and colleagues and by this means, employees develop commitment to their organization (Kim et al., 2017). When we consider teachers, engaged teachers are more likely continue to work in their current school, thus they have OC (Bakker et al., 2003). On the other hand, teachers with relatively less OC generally look for chances to leave the organization (Shirbagi, 2007). Based on these, the following hypothesis was established.

*H3: Teachers’ organizational commitment positively predicts their work engagement.*

#### Emotional Labor, Organizational Commitment, and Work Engagement

Although there is a growing interest in teachers’ emotions, there is not still enough empirical evidence regarding positive emotions of teachers and how positive emotions influence individual and organizational outcomes. The dynamics of teacher engagement also holds great importance as their attitudes and level of engagement have a direct impact on their students (Roth et al., 2007). Teachers’ WE determines the quality of their teaching and the nature of classroom behavior. Therefore, it is crucial to understand the factors that lead higher levels of teacher engagement and its relationship with EL to enhance quality in schools.

Previous research investigated the associations of EL, OC, and WE with burnout (Lapointe et al., 2012; Xin et al., 2017; Han S. S. et al., 2018), self-efficacy, optimism, trust (Agu, 2015; Tösten and Toprak, 2017; Liu and Huang, 2019), compassion, work ethics, leadership (Mauno et al., 2016), organizational support (Xin et al., 2017), job satisfaction (Lin et al., 2020), and well-being (Rusu and Colomeischi, 2020). However, these were mostly carried out in business or hospital contexts. However, the current study aimed to explore the relationships between teachers’ EL, WE, and OC in school contexts. In this way, it aimed to extend the literature on these variables by examining the mediator role of OC on the relationship between WE and EL. Thus, the following hypothesis was suggested.

*H4: Teachers’ organizational commitment mediates the relationship between their emotional labor and work engagement.*

## Materials and Methods

### Model

This study adopted a cross-sectional design (Kothari, 2004), one of the quantitative methods, to investigate the relationships among EL, OC, and WE in schools. These relationships were explored through a structural equation model.

### Sample

The current study was conducted in Sakarya province in Turkey. The participants were reached through convenience sampling method (Huck, 2012) and 429 teachers (from preschool to high school level) voluntarily responded the scales. Before the data analysis, we carried out normality tests which resulted in 57 outliers. Therefore, the main data analysis was conducted on data from 372 participants which can be deemed enough for a structural equation model (SEM) model according to Kline (2009). Of these participants 232 were women (62.4%) and 140 were men (37.6%). While 316 of the participants had an undergraduate degree (85.0%), 56 of them had a graduate degree (15.0%). As for marital status, 301 participants were married (80.9%) and 71 of them were single (19.1%). Lastly, 32 of the participants were working at primary schools (8.6%), 158 at elementary schools (42.5%), 108 at secondary schools (29.0%), and 74 at high schools (19.9%).

### Data Collection Process and Tools

Since the data were collected during COVID-19 pandemic, an online data collection procedure was followed. We prepared an online link (Google Forms) and sent it to the school administrators we are acquainted with. The administrators shared the link with teachers through school WhatsApp groups. The data collection procedure took place between June and November, 2020.

To collect data three different scales which are “Organizational Commitment Scale,” “Utrecht Work Engagement Scale,” and “Emotional Labor in Schools Scale” were used in the study. In the following section detailed information about the scales are presented.

### Emotional Labor in Schools Scale

The scale was developed by Öztürk (2020). The scale has 12 items loading on three dimensions. The first dimension is “emotional effort (7 items),” the second one is “emotional transparency (2 items),” and the last one is “negative emotional transfer (3 items)” which all addresses deep acting of EL. A sample item is as follows: I try to get rid of negative emotions while going to school. The scale is a five-point Likert type scale, and the response range is (1) Totally disagree and (5) Totally agree.

### Organizational Commitment Scale

The scale was developed by Meyer et al. (1993) and adapted into Turkish by Dağlı et al. (2018) for a teacher sample. The scale has 18 items loading on three dimensions. The first dimension is “affective commitment (6 items),” the second one is “continuance commitment (6 items),” and the last dimension is “normative commitment (6 items).” A sample item is as follows: I regard this school’s problems as mine. This is a five-point Likert type scale in which all items are scored on a range from “(1) Strongly disagree” to “(5) Totally agree.”

### Work Engagement Scale

To measure WE level of teachers, Utrecht Work Engagement Scale (Schaufeli et al., 2002) was used. The scale was adapted to Turkish by Atilla-Bal (2009). Later, Eser (2018) confirmed the validity of the scale on a teacher sample. It is a tridimensional scale, which are “vigor (6 items),” “dedication (5 items),” and “absorption (6 items).” A sample item is as follows: “When I get up in the morning, I feel like going to work.” The items are responded on a five-point rating scale ranging from “(1) Strongly disagree” to “(5) Totally agree.”

Within the scope of this study, we checked the internal consistency of the scales through Cronbach’s alpha coefficients. The findings showed that it was 0.71 for EL; 0.84 for OC and 0.92 for Work Engagement Scale which were satisfactory (Büyüköztürk, 2011).

### Data Analysis

First of all, the missing values were detected and there were none of them. Before conducting descriptive analysis and calculating relationships between variables, univariate normality was checked through skewness and kurtosis values. The analysis showed that the data were not normally distributed (Field, 2009). Therefore, we assessed the outliers using boxplots which yielded the exclusion of 57 of them and a normally distributed data (see Table 1). The analysis went on with data from 372 participants. Within the descriptive statistics, we calculated minimum, maximum, mean, and standard deviation of the mean. To reveal the relationships between variables, Pearson correlation coefficients were calculated.

**TABLE 1:** Descriptive statistics and normality assumption.

**Variable**	***N***	**Descriptive**				**Skewness and kurtosis**			
		**Minimum**	**Maximum**	**Mean**	**SD**	**Skewness**	**SE**	**Kurtosis**	**SE**
(1) EL	372	2.75	5.00	3.88	0.43	0.235	0.126	0.464	0.252
(2) OC	372	1.61	5.00	3.42	0.61	−0.085		0.021	
(3) WE	372	3.00	5.00	4.14	0.49	0.136		−0.856	

*EL, emotional labor; OC, organizational commitment; WE, work engagement.*

The hypotheses were tested through SEM, which is a statistical technique allowing the analyst to investigate a series of dependence relationships between variables (Ho, 2006). In SEM we followed the steps suggested by Hair et al. (2014). Actually, these include two stages mainly, the evaluation of the measurement and the structural model. One of the issues that should be addressed in SEM is multivariate normality (Ho, 2006). So, we checked multivariate kurtosis and its critical value, which were 405.361 and 65.833 indicating non-normally multivariate distribution (Bentler, 2006). Thus, bootstrapping (sample 5000; 95% confidence interval) was preferred (Zhao et al., 2010). The basic underlying principle of bootstrapping is that it allows the researchers to create multiple subsamples from an original data base and it does not require multivariate normal distribution (Byrne, 2016).

To test whether there is common method bias problem, we employed Harman’s single factor technique. We loaded all observed variables into an exploratory factor analysis with an unrotated factor solution. The analysis resulted in nine factors explaining nearly 64% of the total variance. The first factor, on the other hand, explained only 27% of the variance which indicates that there is not common method variance problem (Podsakoff et al., 2003).

## Findings

In this section, descriptive statistics and correlations between variables are presented.

Table 1 shows the descriptive statistics and the results of normality tests. The mean is *M* = 3.88 (SD = 0.43) for EL, *M* = 3.42 (SD = 0.61) for OC and *M* = 4.14 (SD = 0.49) for WE. Considering these findings, we can say that teachers’ self-perceptions of EL, OC, and WE are above average and relatively high.

Table 2 displays correlations between variables. The relationship between EL and OC is (*r* = 0.294; *p* < 0.01); EL and WE (*r* = 0.482; *p* < 0.01) and OC and WE (*r* = 0.479; *p* < 0.01). These findings indicated statistically significant positive and moderate level relationships between variables.

**TABLE 2:** Correlations between variables.

**Variables**	**EL**	**OC**	**WE**
EL	1.00		
OC	0.294**	1.00	
WE	0.482**	0.479**	1.00

**p < 0.01.

### Findings on Measurement and Structural Model

**FIGURE 1:**
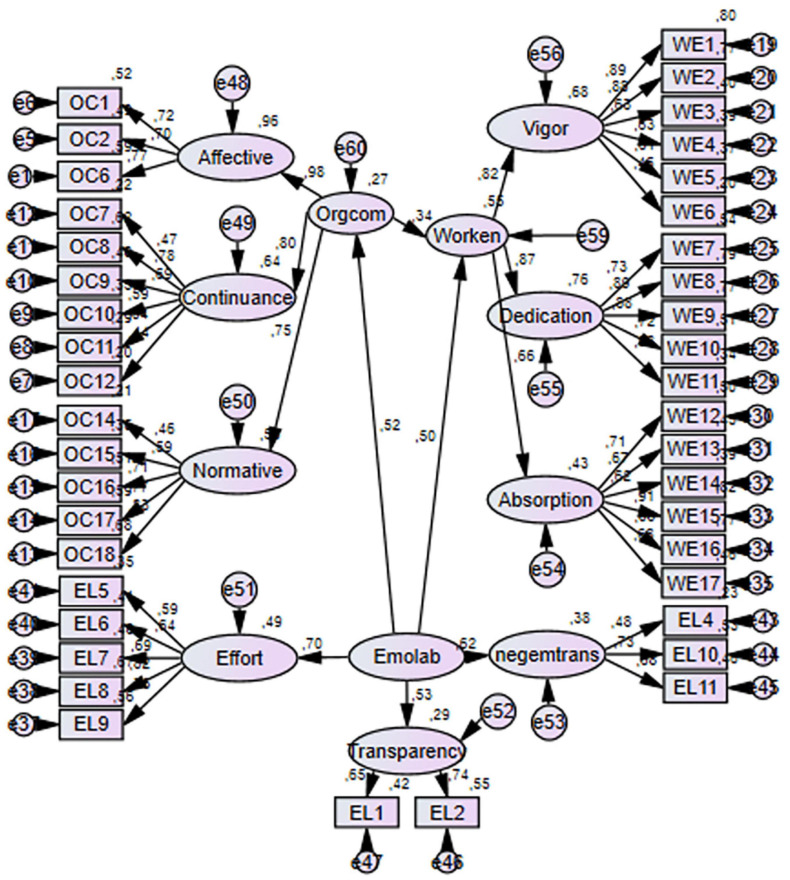
Structural model.

As stated above we followed a two-step approach in the analysis. First of all, we evaluated the measurement model in which all the observed variables were in the model. In the first analysis, the fit indices of the model did not satisfy the cutoff values in the literature and due to low factor loadings (OC3 = 0.187; OC4 = 0.348; OC5 = 0.371; OC12 = 0.443; EL3 = 0.352; EL12 = 0.415) we discarded some items. We reran the analysis and the fit indices emerged as follows (*x*^2^/df = 2.69; *p* = 0.00; CFI = 0.83; TLI = 0.81; RMSEA; 0.07; SRMR = 0.08). Considering the number of observed variables and the sample size, the fit indices can be deemed satisfactory (Sharma et al., 2005; Hair et al., 2014). Ensuring the validity of the measurement model, we tested the structural relationships between variables. The model is shown in Figure 1 below.

The structural relationships between variables are displayed in Figure 1. First of all, we checked the fit indices of the model. They emerged as following (*x*^2^/df = 2.69; *p* = 0.00; CFI = 0.83; TLI = 0.81; RMSEA; 0.07; SRMR = 0.08) which were the same as in the measurement model. The results of bootstrap analysis provided evidence to support all the hypotheses suggested. As can be seen in Table 3 below, EL predicted WE (β = 0.502; *p* = 0.002) and OC (β = 0.519; *p* = 0.001) significantly. OC, on the other hand, predicted WE significantly (β = 0.345; *p* = 0.045). Lastly, the indirect effect of EL on WE through OC was significant (β = 0.179; *p* = 0.002) and upper-lower bounds did not include “0” (LB = 0.083; UB = 0.222). Both the direct and indirect effects are significant and in the same direction which indicates a complementary mediation (Zhao et al., 2010). On the other hand, the effect size for indirect effect was calculated using the formula ab / (ab + c1) (Miočević et al., 2018) which emerged as 0.261. It can be concluded that it has a medium effect size (Cohen, 1988). The findings are presented below in Table 3.

**TABLE 3:** Standardized direct, indirect, and total effects.

**Paths**	**β**	**SE**	***t***	**Bootstrap 5000 times 95% CI**		***p***	**Total effect**
				**Lower bound**	**Upper bound**		
EL→WE (H_1_)	0.502	0.157	5.001	0.190	0.800	0.002	0.681
EL→OC (H_2_)	0.519	0.132	5.715	0.245	0.734	0.001	0.519
OC→WE (H_3_)	0.345	0.152	4.042	0.021	0.557	0.045	0.345
EL→OC →WE (H_4_)	0.179	0.054	–	0.083	0.222	0.002	0.681

*Standardized direct and indirect effects = 95% CI does not include zero.*

## Results and Discussion

This study investigated the mediating role of OC on the relationship between teachers’ EL and WE. It also explored the direct relationships among these variables. In this sense, the first hypothesis of the study was that teachers’ EL significantly predicts their WE. The findings confirmed the hypothesis which means that higher levels of EL results in higher levels of WE for teachers. On the other hand, there are inconsistent findings in literature regarding this relationship which was investigated on different samples such as private sector workers (Chan, 2009), customer service employees (Pelosi, 2015), nurses (Mauno et al., 2016; Han S. S. et al., 2018), school foodservice employees (Heo and Lee, 2015), incubation centre managers (Kim et al., 2015), service sector employees (Öngöre, 2019), retail bank and insurance company employees (Yoo, 2016), salespeople (Yoo and Jeong, 2017), and full time workers who participated in a management training course (Wang et al., 2010). There are also studies conducted on teachers (Saleem et al., 2018; Shukla and Pandey, 2019; Çarıkcı, 2020; Košir et al., 2020). While some of these studies show that EL is positively associated with WE (Chan, 2009), some others (Mauno et al., 2016; Saleem et al., 2018; Shukla and Pandey, 2019) show that there is a negative association between them (Mauno et al., 2016; Saleem et al., 2018; Shukla and Pandey, 2019). Considering the relationships in terms of sub-dimensions, some studies (Wang et al., 2010; Heo and Lee, 2015; Kim et al., 2015; Pelosi, 2015; Yoo, 2016; Yoo and Jeong, 2017; Han S. S. et al., 2018; Öngöre, 2019; Košir et al., 2020) indicate that deep acting and naturally felt emotions are positively associated with WE, while according to some others (Wang et al., 2010; Pelosi, 2015; Yoo, 2016; Yoo and Jeong, 2017; Han S. S. et al., 2018; Öngöre, 2019; Çarıkcı, 2020; Košir et al., 2020) surface acting is negatively associated with it. On the other hand, Mróz and Kaleta (2016) found that there is not a significant relationship between these two variables. Based on these, it is can be said that the current study has both consistent and conflicting findings with the previous research. It can also be said that further research is needed on the relationship between teachers’ EL and WE to have a more robust insight into this relationship. In this sense, this study contributed to the existing literature by providing empirical evidence on the effect of teachers’ EL on WE. So, it is suggested that if schools wish more engaged teachers, they should create an atmosphere in which teachers can display EL.

The second hypothesis of the study suggested that there was a positive association between EL and OC which was confirmed by the findings. When teachers can exhibit emotional effort and transparency and transfer their negative emotions, they become more committed to the school. However, there are inconsistent findings in the literature on both teacher (Isenbarger and Zembylas, 2006; Güler, 2018; Deliveli and Kıral, 2020; Ogunsola et al., 2020; Zheng et al., 2020) and other samples such as health employees (Şenel and Aydoğan, 2020), hotel employees (Büyükbeşe and Aslan, 2019), nurses (Yang and Chang, 2008; Han S. S. et al., 2018), and IT workers (Kim and Yang, 2018). While some of these studies suggest a positive association (Isenbarger and Zembylas, 2006; Güler, 2018; Deliveli and Kıral, 2020; Şenel and Aydoğan, 2020), some others reveal that there is a negative relationship between EL and OC (Han S. L. et al., 2018; Büyükbeşe and Aslan, 2019). Considering the relationships in terms of the dimensions, Zheng et al. (2020) suggested that deep acting is positively associated with commitment and surface acting has a negative effect on it, while Ogunsola et al. (2020) found that both surface and deep acting has a negative effect on OC. On the other hand, Yang and Chang (2008) indicated that surface acting has a significantly negative relationship with OC while deep acting does not have a significant effect on it. On the contrary, Kim and Yang (2018) demonstrated that the deep acting has a significant relationship with OC while surface acting is not related to it. As stated above previous literature suggests inconsistent findings regarding the relationship between EL and OC and the studies focusing on teachers are insufficient. Considering this gap, the current study extended the existing literature especially in terms of educational organizations. In this sense, this study shows how educational organizations take care of their teachers’ EL since it affects teachers’ OC. If these organizations ask the teachers to show commitment to them, then they help these teachers to increase their EL. So, the motto is “to have more committed teachers, schools need to help teachers manage their emotions.”

The third hypothesis of the study suggested that teachers’ OC positively predicts their WE. The findings confirmed the hypothesis which means that the higher teachers are committed to their school the higher they will be engaged to their work. The studies conducted on teachers (Çağrı San and Tok, 2017; Pieters and Auanga, 2018) and other samples such as banking employees (Adi and Fithriana, 2020), private and public sector employees (Agyemang and Ofei, 2013), undergraduate students (Babcock-Roberson and Strickland, 2010), airline companies (Li et al., 2010), National Revenue Administration employees (Peplińska et al., 2020), also put forward consistent findings with this study, while food processing plant employees (Gota, 2017) has found that WE is negatively associated with affective commitment. Based on these findings, it can be said that this study contributed to the literature by proving that WE is up to OC. So, if educational organizations can make their teachers more committed to them, then it will be more likely that these teachers will engage their works much more. In brief, to have more engaged teachers, schools should foster teachers’ OC.

The last hypothesis of the study dealt with the indirect effect of EL on WE through OC. The findings indicated that commitment mediated the relationship between EL and engagement. In other words, EL increases OC which in turn has a positive effect on WE of teachers. Although at least in scope of the current study we could not reach a study investigating the relationships among these three variables, there are studies on EL, OC, and some other variables. For example, they were associated with burnout (Hakanen et al., 2006; Lapointe et al., 2012; Yılmaz et al., 2015; Xin et al., 2017; Han S. S. et al., 2018; Yin et al., 2019), self-efficacy, optimism, trust (Agu, 2015; Tösten and Toprak, 2017; Liu and Huang, 2019), compassion, work ethics, leadership (Mauno et al., 2016), organizational support (Xin et al., 2017), job satisfaction (Lin et al., 2020), political skills (Bostancı, 2020), well-being (Rusu and Colomeischi, 2020), and organizational citizenship behavior (Cheung and Lun, 2015). Drawing on the findings of the current study, we can say that OC plays a significant role on the relationship between EL and WE of teachers. For this reason, this study shows that if educational organizations aim to reach WE for their teachers, then they should know that the first step goes through EL, while the second one through OC. For this reason, a three-step roadmap is suggested to educational organizations. Firstly, they should take action to provide their teachers to put in their emotional labol more; secondly, the teachers will show more commitment to their organizations. After all is said and done, the teachers will engage their works much more which is the final destination in this journey.

### Conclusion and Suggestions

This study can be the first one which addresses EL, OC, and WE for teachers. In this context, it can be said that it is valuable for both researchers and future studies since the obtained results can contribute new knowledge to the field. In this way, this study extended the literature on EL, OC, and WE. The most significant contribution of the current study is that it provided evidence that OC of teachers can mediate the relationship between their EL and WE. When teachers exert emotional effort, transfer their negative emotions, and exhibit their emotions transparently, they become more committed to school and in turn they become more engaged in their work. This underlines the importance of emotion regulation for teachers. School administrators should be aware of importance of teachers’ emotions and create an environment in which teachers can display their emotions. They should also consider that EL and OC explain WE. In this way, they can know how to proceed for engaging teachers to their works and to implement the practical effect on work life. Besides these points, it is suggested that more studies should be made to use this model on teacher samples. Considering the literature, it can be said that there is a serious need on this suggestion.

### Limitations and Implications for Further Research

This study is not without some limitations. Firstly, the study employed a cross-sectional design which does not provide cause-effect relationships. Further research may employ longitudinal design to reveal causality. Secondly, the findings of the current study are based on teachers’ self-perceptions which may cause social desirability bias (Rosenman and Tennekoon, 2011). Thirdly, we reached the participants through convenience sampling which may cause the problem of generalizability. However, this is an internal validity study which aims to test a structural model not to generalize the findings to a target population. Fourthly, we tested a simple mediation model. The findings indicated complementary mediation which means an incomplete theoretical framework (Zhao et al., 2010). Fifthly, this study used the EL scale developed by Öztürk (2020) and addressing EL based on deep acting. For this reason, the hypothesis of the study was created accordingly. Further studies can choose a different scale which runs all the theoretical structure of EL. Further studies may consider extending the model integrating new mediators or moderators such as tenure or age. Lastly, the current study was conducted only one city in Turkey. Cross-cultural validation of the model can be considered in further studies.

## Data Availability Statement

The raw data supporting the conclusions of this article will be made available by the authors, without undue reservation.

## Ethics Statement

The studies involving human participants were reviewed and approved by the Sakarya University Ethics Committee with the decision number E-61923333-050.99 and subject 29/35. Only the volunteered participants participated in this study.

## Author Contributions

GS-G devised the research idea, developed the research model, performed the results, discussion, recommendations, limitations, revised and improved the introduction and method parts for the manuscript, and arranged the last version of the manuscript. MB ran the data collecting process, wrote the introduction part, and controlled the other parts in terms of language and contextual check for the manuscript. İL wrote the method part, ran the analytic calculations, limitations, and implications, and checked for the literature and discussion part. All authors contributed to the article and approved the submitted version.

## Conflict of Interest

The authors declare that the research was conducted in the absence of any commercial or financial relationships that could be construed as a potential conflict of interest.
